# Shoulder Kinematics and Spatial Pattern of Trapezius Electromyographic Activity in Real and Virtual Environments

**DOI:** 10.1371/journal.pone.0116211

**Published:** 2015-03-13

**Authors:** Afshin Samani, Charles Pontonnier, Georges Dumont, Pascal Madeleine

**Affiliations:** 1 Laboratory for Ergonomics and Work-related Disorders, Center for Sensory-Motor Interaction (SMI), Department of Health Science and Technology, Aalborg University, Fredrik Bajers Vej 7 D-3, 9220, Aalborg East, Denmark; 2 Centre de Recherche des Ecoles de Coëtquidan, Ecoles Militaires de Saint-Cyr Coëtquidan, 56 381, Guer, France; 3 MimeTIC, IRISA/INRIA Centre de Bretagne, Campus de Beaulieu, 35042, Rennes, France; 4 ENS Rennes, Campus de Ker Lann, 35170, Bruz, France; VU University Amsterdam, NETHERLANDS

## Abstract

The design of an industrial workstation tends to include ergonomic assessment steps based on a digital mock-up and a virtual reality setup. Lack of interaction and system fidelity is often reported as a main issue in such virtual reality applications. This limitation is a crucial issue as thorough ergonomic analysis is required for an investigation of the biomechanics. In the current study, we investigated the biomechanical responses of the shoulder joint in a simulated assembly task for comparison with the biomechanical responses in virtual environments. Sixteen male healthy novice subjects performed the task on three different platforms: real (RE), virtual (VE), and virtual environment with force feedback (VEF) with low and high precision demands. The subjects repeated the task 12 times (i.e., 12 cycles). High density electromyography from the upper trapezius and rotation angles of the shoulder joint were recorded and split into the cycles. The angular trajectories and velocity profiles of the shoulder joint angles over a cycle were computed in 3D. The inter-subject similarity in terms of normalized mutual information on kinematics and electromyography was investigated. Compared with RE the task in VE and VEF was characterized by lower kinematic maxima. The inter-subject similarity in RE compared with intra-subject similarity across the platforms was lower in terms of movement trajectories and greater in terms of trapezius muscle activation. The precision demand resulted in lower inter- and intra-subject similarity across platforms. The proposed approach identifies biomechanical differences in the shoulder joint in both VE and VEF compared with the RE platform, but these differences are less marked in VE mostly due to technical limitations of co-localizing the force feedback system in the VEF platform.

## Introduction

The use of virtual reality (VR) in relation to human factors and ergonomics is of certain interest to professionals involved in the design of technological interfaces, the improvement of work efficiency and the overall reduction of costs [1,2]. Digital human modeling and virtual human simulations are examples of such applications [3]. In particular the augmentation of VR setups with motion capture systems allows the calculation of the biomechanical attributes of a task. In turn these attributes can be utilized to analyze the physical risk factors that can lead to the discomfort and pain characterizing musculoskeletal disorders [4].

VR systems have advantages such as availability, safety and data provision. However, these systems still suffer from drawbacks related to the adopted technological solutions [2]. For instance, the level of the fidelity of VR to a similar real environment should be thoroughly addressed [5]. System and interaction fidelities as defined in [6] are of particular importance as they directly influence the motion and the biomechanical load. To our best knowledge only a few studies have assessed the biomechanics of the shoulder girdle in VR environments [7,8]. Despite fair correlations between VR and real biomechanical metrics, a recent study has pointed out considerable discrepancies between VR and real environments [9,10]. Particularly the range of movement between a VR and a real box-lifting task has been found to be comparable whereas significant differences have been reported in terms of movement velocity and acceleration [8]. In a recent study [7] we also found that the potential risks posed by posture for neck-shoulder musculoskeletal disorders are different in real and virtual environments. In the latter study, postural risk factors were investigated over all segments of the upper extremity. However, due to the complex structure of the shoulder joint and the high prevalence of disorders in that region and its large range of motion, special attention has been drawn to movement patterns of this joint [11]. Some studies also suggest that the control of pointing movement can be decomposed into shoulder-elbow and wrist components. They conclude that shoulder flexion and elbow extension are independent of the wrist angular motion [12]. This study kept a focus on a detailed investigation of the shoulder joint in tasks performed in VR environments.

A thorough assessment of the biomechanical response in VR platforms may require more than a mere comparison of the kinematic properties of movements. Surface electromyography (EMG) analysis can complement findings on the kinematic aspects of movements [13,14]. In our previous study we found a good correlation between a real and a virtual setup in terms of muscular activity in the upper extremities [7]. However, conventional bipolar EMG has a poor spatial representation of muscle activity [15]. As an alternative, high-density EMG (HD-EMG) from the upper trapezius creates the spatial pattern of muscular activity and thereby improves the physiological representation of the muscle being studied [16,17]. That being so, changes in the spatial pattern of the upper trapezius EMG activity have been reported in the presence of, for example, fatigue and pain [16,18].

Apart from the working environment, task attributes such as precision demands also affect the EMG activity of the upper trapezius muscle [19]. The biomechanical responses in VR environments could in turn be affected by the task attributes [5,20], although this has not yet been thoroughly investigated.

The current study compares the shoulder kinematics and the spatial pattern of the trapezius activation during performance of a simulated real task and a similar virtual task with and without haptic force feedback. In ergonomics the extent of intra-subject variability of the exposure outcomes across the two different conditions has been compared with inter-subject variability within one of the conditions and this comparison has provided a contrast index between the two conditions [21,22]. Therefore, if the intra-subject variability across the conditions is greater than the inter-subject variability within a task, the two conditions will be seen as different working conditions. Along with such an approach one can apply methods such as cross-correlation and normalized mutual information (NMI) to quantify the similarity (the opposite of the concept of variability) of the biomechanical response of subjects in different conditions and compare the inter- and intra-subject similarity [23]. NMI as a similarity index has been established and has a broad application in fields such as image processing [24] and biomedical signal processing [23]. In the context of biomechanics, mutual information has also been used to study the nonlinear properties of joint angle trajectories during gait [25]. We hypothesized that the intra-subject similarity of biomechanical response across real and virtual environments is comparable with inter-subject similarity within the real environment. The adopted approach can facilitate the assessment of improvements in VR-based solutions used for evaluating physical risk factors at work.

## Methods

Sixteen male participants (aged 26.5±2.8 years; height 178.4±6:5 cm; body mass 70.2±9.2 kg) took part in the present study. The study population was the same as in our recent study [7]. No participants reported any history of neck-shoulder disorders and all were novices in VR (average experience of 1.4±0.5 on a five-point scale ranging from “1: Novice” to “5: Expert”). The study was conducted in accordance with the Helsinki Declaration and was approved by the local ethics committee (Den Videnskabsetiske Komité for Region Nordjylland, N-20120036). The participants signed an informed written consent form prior to the experiment.

### Experimental Procedure

The participants performed a simulated task on three different platforms, i.e., a real (RE), a virtual (VE) and a virtual environment with haptic force feedback platforms (VEF). The inclusion of force feedback is supposed to yield to a higher level of fidelity in VR environments [9]. The task was a simplified assembly task and consisted of target reaching, object manipulation and sorting. Further, the investigated task was performed while the participants were standing.

**Fig 1:**
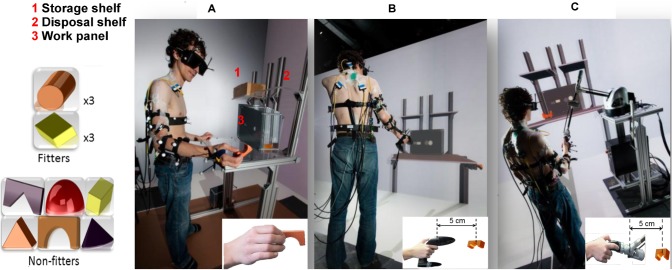
Illustration of the work place within the three platforms, i.e., a) real, b) virtual and c) virtual with force feedback. The participants stood in front of a work table adjusted to their elbow height and had to grasp an object from the storage shelf with the right hand. The participants had to pass the “fitter” objects through the work panel and laid the “non-fitter” objects on the disposal shelf. Consent to publication was obtained from the participant seen in the figure.

The RE platform consisted of storage and disposal shelves, a work panel, and twelve wooden objects (see Fig. 1). The work panel had two holes with different cross-sectional contours which could accommodate some of the objects (“*fitter*”) whereas the other objects (“*non-fitter*”) could not pass through any of the holes. The work panel was located on a table at elbow height in agreement with recommendations for light work [26]. The storage and disposal shelves were located 40 cm above the table surface and 16 cm to the left and right of the work panel center, respectively.

Within each of the platforms the participants had to move their right hand towards the objects on the storage shelf and grasp one after receiving a verbal let-go signal. The participants had to pass the “*fitter*” objects through the proper hole in the work panel. In contrast they had to place the “*non-fitter*” objects on the disposal shelf. Before the recording on each platform the participants were familiarized with the task for at least 5 min.

Within each of the platforms the participants had to work with six “*fitter*” and six “*non-fitter*” objects in total. Handling of “*fitter*” objects demanded a higher precision than handling of “*non-fitter*” objects. The order of the platforms and object types (“*fitter*” and “*non-fitter*”) was randomized. The participants took a 2 min rest prior to switching between the platforms.

The VE and VEF were designed to resemble the RE but we acknowledge the limitations mentioned in our previous work (see also the discussion section) [7]. An overview of the hardware design and the numerical pipeline can be found in another previous work [27]. The 3D representation of the workstation and the work panel were derived from the digital mock-ups used to fabricate the RE. The virtual table height was also visually adjusted with respect to the elbow height of the participants. The virtual system used a high-resolution stereoscopic immersion room including a wall (front screen) and a floor (vertical wall: 9.6m×3.1m, 6240×2016 pixels, eight Barco NW12 projectors, BARCO Inc., USA; floor: 9.6m×2.88m, 3500×1050 pixels, three Barco Galaxy 7 projectors, BARCO Inc., USA). 3D glasses (ActiveEyes-Pro, Volfoni, SAS, France) tracked by a 360° tracking system with 16 ART infrared cameras (Advanced Realtime Tracking GmbH, Germany) were used. The distributed architecture of the room was based on the framework described in [28]. The interaction with the VE was done with a wireless stick (Flystick2, Advanced Realtime Tracking GmbH, Germany) co-localized with the virtual scene. The flystick allowed navigation of the 3D immersive environment and camera tracking to obtain the position of the object as well as the transmission of the user command by means of a trigger button. The VEF was very similar to VE except that instead of the flystick the participant had to interact with the VR environment using a six-degree of freedom haptic device (Virtuose, Haption SA, France) co-localized with the virtual scene. The device was chosen for its large work volume (0.7 m x 0.5 m x 0.5 m) and its ability to render a range of forces close to the real ones (maximal continuous force of 10N). The participants performed the task with wooden objects, each weighting 20 g, the flystick (280 g) and the haptic device producing a simulated force of 0.2 N in RE, VE, and VEF, respectively. For VE and VEF platforms, the physics of the scene were simulated with the Bullet Physics Library (bulletphysics.org). Simplified forms of non-convex objects were separately designed for the physics simulation. Both the virtual coordinates of Flystick and Virtuose devices were linked to the real coordinates by means of a standard proportional derivative control scheme for positions and a suboptimal control scheme with a quadratic cost for rotations [29]. Virtual gains were set to minimize undesirable collision effects such as jumps, vibrations and instability on the virtual objects [29].

Grasping of the objects in VE and VEF was different from RE (see Fig. 1). It was done by a trigger button actuated with the forefinger and the thumb in VE and VEF, respectively. The object was placed 5 cm above the center of the flystick or the haptic device to provide a proper visual interface for the participants. The offset was a relative offset and was not affected by the motion of the participants. This difference may have some effect on the kinematic patterns of movement. However, this offset represented only 8 to 16% of the range of movement, i.e., 32 cm (in “*non-fitter*” cycles) and 60 cm (in “*fitter*” cycles). Further, only one active and visible object was displayed on the storage shelf at the time of grasping within the VE and VEF platforms.

### Data Recordings

The orientations of the arm in 3D, i.e., shoulder flexion (SF), internal rotation (SI), and shoulder abduction (SA), were tracked by six dedicated AR-Tracking targets, sampled at 60 Hz. As the tracking outputs consisted of both the positions and the orientations of each segment relative to the immersive room reference frame, the relative rotation matrix describing the motion of the shoulder relative to the upper body (shoulder-upper body matrix) was obtained by multiplying the inverse upper body-reference matrix by the shoulder-reference matrix. The identification of the joint coordinates was performed from the rotation matrix on the basis of a simple spherical joint representing the shoulder [30,31]. The sequence of rotation angles over the time of each cycle was termed an angle trajectory. The rotation angles were reported with respect to a reference standing position with both arms along the body.

The tracking system was able to track the hand position with a dedicated cluster of optical markers. The markers were attached to the hand of the participants throughout the whole experiment.

HD-EMG signals were recorded with a semi-disposable adhesive grid of 64 electrodes (LISiN-Spes Medica, Italy, model ELSCH064R3S). The grid consisted of 13 rows and 5 columns of electrodes (2-mm diameter, 8-mm inter-electrode distance in both directions) with a missing electrode at the upper left corner serving as the origin of the coordinate system to define the electrode location [18]. The 64-electrode grid was then placed on the upper trapezius muscle with the fourth row aligned with the C7-acromion line, parallel to the muscle fiber direction. The lateral edge of the grid was 10 mm medial to the mid-point of a line between C7 and the acromion.

The silver-silver chloride electrode surfaces in the grid were separated from the skin by a small cavity (∼1-mm thick) filled with a conducting paste. The EMG signals were differentially amplified 2000 times (128-channel surface EMG amplifier, EMG-USB, LISiN-OT Bioelectronica, Turin, Italy; 3-dB bandwidth, 10 to 500 Hz), sampled at 2048 Hz, and A/D converted in 12 bits. A reference electrode was placed on the C7 vertebra. Bipolar signals were computed along the fiber direction, and thus 51 bipolar derivations were obtained and arranged in nodes of a 13×4 grid.

As a subjective evaluation of the tasks, the subjects scored the rate of perceived exertion around the shoulder area between “0: no exertion” and “10: maximal exertion” after finishing the task in each platform. Moreover, the cycle time was also registered (see above). The cycle time was defined as the averaged time elapsed between the beginning and the end of the cycles within each platform and object type.

### Data Analysis

The participant movement in each platform was divided into 12 cycles corresponding to the cycles described above. The onset and offset of the cycles were determined from the time of moving the hand towards the object until the object was passed through the work panel (“*fitter*” cycles) or laid on the disposal shelf (“*non-fitter*” cycles). The reaching times were determined using the kinematic trajectories.

**Fig 2:**
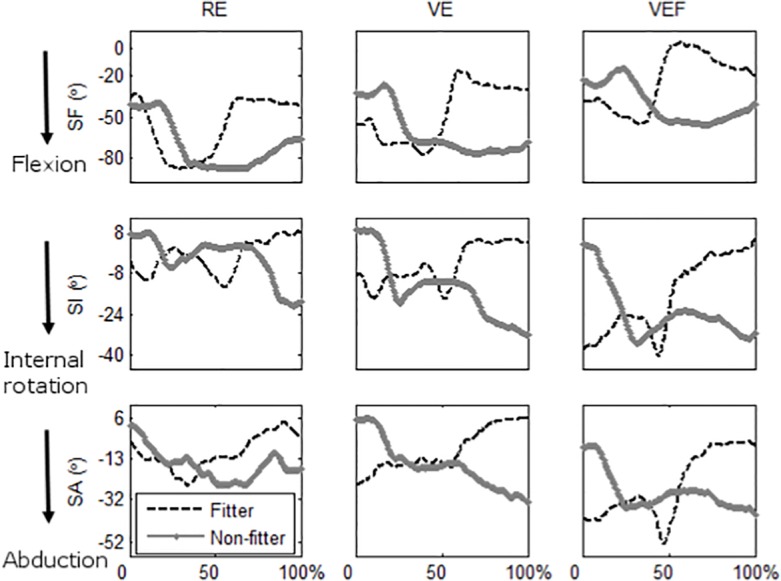
An example of the shoulder flexion (SF), internal rotation (SI) and abduction (SA) angle across a 0 to 100% timeline of a cycle for “fitter” (dashed black) and “non-fitter” (solid gray) objects and “Real” (RE), “Virtual” (VE) and “Virtual with force feedback” (VEF) platforms. For “fitter” objects, the task requires consecutive shoulder flexion and extension to grasp an object and bring it down to pass it through the work panel. The shoulder flexion and extension were concomitant with a short burst of internal rotation and the shoulder was kept adducted while the object was passed through the work panel. For “non-fitter” objects, the shoulder was flexed to grasp an object and then it was kept flexed until the object was laid on the disposal shelf. The shoulder was internally rotated and abducted during approaching and after grasping the object.

The velocities of the 3D angular trajectories were computed by numerical differentiation after smoothing the trajectories (bidirectional second order Butterworth low pass at 8 Hz [32]). The maximum values of instantaneous angle and velocity were obtained in 3D. The directions consisted of flexion-extension for SF (SF-Flx and SF-Ext), internal-external rotations for SI (SI-Int and SI-Ext), and abduction-adduction for SA (SA-Abd and SA-Add). For example, the maximal deviation of the shoulder from the neutral posture in the direction of shoulder flexion was considered shoulder flexion maximum and the minimal deviation was considered shoulder extension maximum. Fig. 2 shows an example of SF, SI, and SA angle trajectories over a typical cycle for *fitter* and *non-fitter* objects. The mean of the maximum values of the angular trajectories and their velocity profiles were extracted across the cycles for each participant, object type and platform [8]. This is relevant for ergonomic risk indices such as the rapid upper limb assessment [33] in agreement with known physical risk factors [34]. Subsequently, the angular trajectories and velocity profiles were interpolated (200 samples represented the trajectories across one cycle). The trajectories were sorted based on the object type and the target location on the work panel (the latter only for “*fitter*” cycles). Then the trajectories were concatenated (6 cycles) to obtain a sequence of cycles performed in each of the platforms for each participant and object type.

For each participant (*S_i_*) the cross correlation coefficient (CC), NMI, and the root mean square of the difference (RMS. Diff) were computed between the concatenated sequence of angular trajectories and velocity profiles within VE and VEF on one hand and the corresponding trajectory within RE on the other hand, i.e., *NMI*(*RE_S_i__,VE_S_i__*) and *NMI*(*RE_S_i__,VEF_S_i__*), respectively. CC and NMI are two indices of the similarity of the two trajectories while RMS.Diff reflects the difference between the two indices. CC and RMS.Diff were utilized as conventional indices of similarity and variation, respectively, whereas the NMI is an index of nonlinear similarity and measures the amount of shared information between two time series [35]. Thus, it was used to quantify the similarity of the spatial pattern of shoulder kinematics. In comparison with other indices of similarity, such as cross-correlation coefficient, NMI encompasses both linear and non-linear correlations between two signals [36] (see the Appendix for further details). The application of non-linear indices such as NMI allows a better understanding of complex systems in VR [37]. NMI varies between “0: no common information” and “1: completely identical information.” Thus, the computed NMI represented an intra-subject similarity index across the virtual platforms.

For each object type in RE a *representative trajectory* was obtained by taking the median of the concatenated angular trajectories and velocity profiles (*S¯*). The NMI between the *representative trajectory/ profile* and the corresponding kinematic trajectories/ profiles provides an index of inter-subject similarity in RE, i.e., *NMI*(*RE_S_i__,RE_S¯_*). This approach is inspired by clustering algorithms such as K-medians [38]. The NMI values were termed SF-NMI, SI-NMI, and SA-NMI for shoulder flexion, internal rotation, and abduction angles, respectively. Similarly, SF-Vlc-NMI, SI-Vlc-NMI, and SA-Vlc-NMI denoted the NMI of the velocity profiles.

A linear envelope of EMG (low-pass filtering with a cut-off frequency of 8 Hz) was computed across one cycle. All the EMG envelopes were interpolated over time and 20 time points represented the temporal changes of the muscular activity across one cycle.

After alignment of the EMG maps [39] in RE a representative EMG map was computed as the median of the maps across the subjects at each node of the EMG grid and each time point. Then, the NMI was calculated as an index of similarity between two images at each time point [40]. Using a similar approach as described for kinematic trajectories, we computed the NMI between the EMG maps of each participant and the representative EMG map at each time point. The computed NMIs were then averaged across time points. This calculation provides an index of inter-subject similarity in RE. As an index of intra-subject similarity within the different platforms, NMI was computed between the EMG maps recorded in VE and VEF and the corresponding EMG map recorded in RE. The NMI between the EMG maps was denoted by HD-NMI.

### Statistics

The number of subjects was initially determined for three repeated measures (corresponding to the three platforms) with a 0.75 correlation between them and with a medium effect size (η = 0.25), confidence level (α = 0.05), and desired power (80%) (G*Power 3.1.4 [41]). A linear mixed model (LMM) fitting the outcome measures, i.e., rate of perceived exertion, the maximum of angular trajectories, velocity profiles, and their corresponding CC, NMI, RMS. Diff, and NMI of EMG maps, was used to investigate and compare the biomechanical responses within the three platforms. To find the most parsimonious and the best fit model a “top-down” modeling strategy [42] was applied. Platforms (RE, VE and VEF) and object types (“*fitter*” and “*non-fitter*”) were introduced as within-subject factors of the LMM. Additionally, a repeated factor associated with the platforms was added to the model to allow for residuals with unequal variance at each level of the platforms. When a significant effect was observed, a Bonferroni adjustment was performed for a pairwise comparison. The following results were achieved for all 16 subjects although 6 subjects could not perform the task within the force feedback platform (hardware issue). However, LMM is capable of handling such unbalanced data sets [42]. In all tests, P<0.05 was considered significant. Mean values (standard error) were reported. The residual of the model was checked for normality (Kolmogorov-Smirnov test) and in cases where normality was violated the LMM was applied to the rank transformed data [43]. Since the LMM was associated with multiple fixed and random factors, the statistical test may have suffered from low power. Thus, we only discuss the significant effects to avoid type II errors.

To achieve an empirical estimation of the precision of the computed NMI of the concatenated sequence of angular trajectories and angular velocity profiles, a bootstrapping resampling technique [44] was used by permuting the order of cycles in the concatenated sequence. We carried out this procedure for 16 (same as number of participants) randomly selected permutations of the cycles in the concatenated sequence. The NMI was computed as described above for the concatenated sequence of cycles. Then between and within (between-permutations) subject variance components of the computed NMI were computed using a one-way random effect model. The variance components were derived using the restricted maximum likelihood algorithm (SPSS 22.0, SPSS Inc., Chicago, IL, USA).

## Results

### Comparison of the Platforms

**Fig 3:**
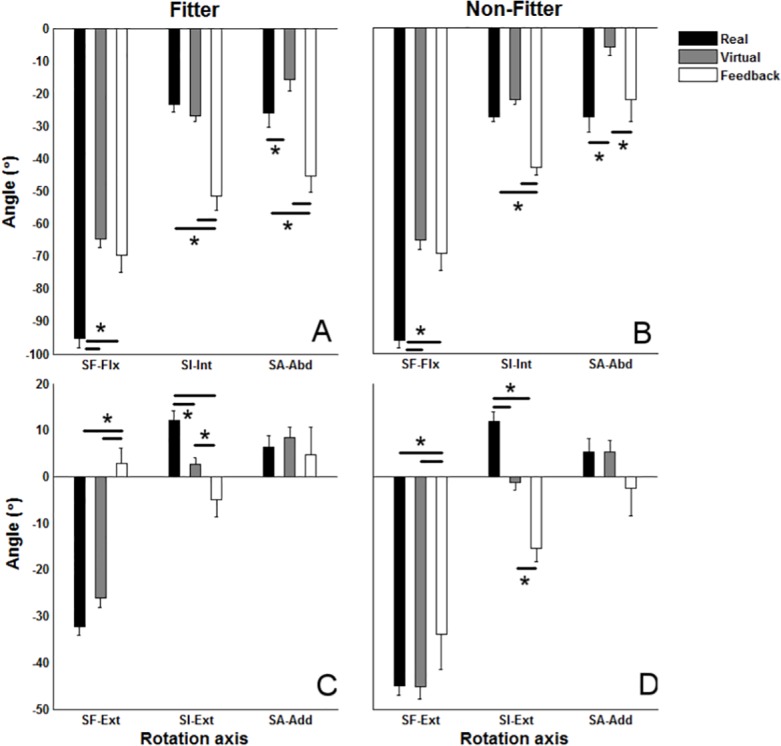
The mean and standard error of maximum of angle trajectories of shoulder flexion (SF), internal rotation (SI) and abduction (SA) across participants and cycles. The figures in each column illustrate the outcomes for each of the object types (“fitter” and “non-fitter”). SF, SI and SA stand for shoulder flexion, internal rotation and abduction, respectively. SF-Flx and SF-Ext stand for maximum flexion and extension of SF angle, SI-Int and SI-Ext stand for maximum internal and external rotation of SI angle, and SA-Abd and SA-Add stand for maximum abduction and adduction of SA angle. * indicate a significant pairwise comparison between Real, Virtual and Virtual environment with feedback (Feedback) (p<0.05).

**Fig 4:**
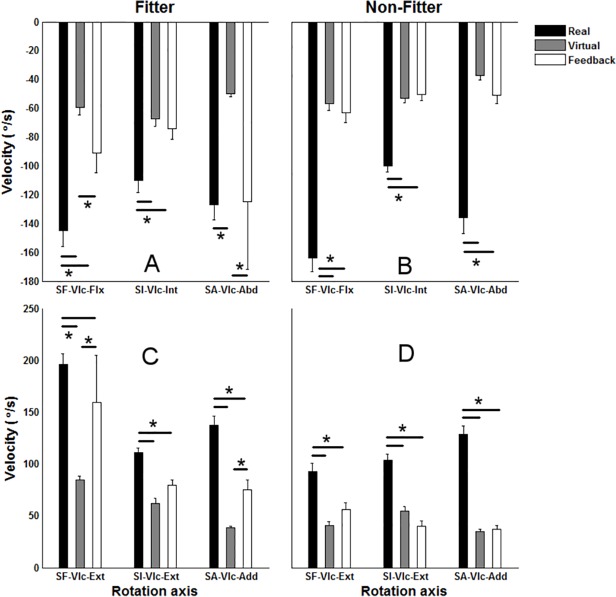
The mean and standard error of maximum of angular velocity profiles of shoulder flexion (SF), internal rotation (SI) and abduction (SA) across participants and cycles. The figures in each column illustrate the outcomes for each of the object types (“fitter” and “non-fitter”). SF, SI and SA stand for shoulder flexion, internal rotation and abduction respectively. SF-Vlc-Flx and SF-Vlc-Ext stand for maximum angular velocity in flexion and extension of shoulder, SI-Vlc-Int and SI-Vlc-Ext stand for maximum internal and external rotation of shoulder, and SA-Vlc-Abd and SA-Vlc-Add stand for maximum abduction and adduction of shoulder. * indicate a significant pairwise comparison between Real, Virtual and Virtual environment with feedback (Feedback) (p<0.05).

The interaction of the platform and the object type (platform× object type) had a significant effect on all angle and angular velocity maxima (P<0.02 in all cases), but a main effect of the platform (P<0.001) and the object (P = 0.003) type was found on SF-Flx and the angular velocity of SI-Int. For the “*fitter”* objects the angular velocity maxima in VEF and RE were generally closer to each other compared with those in RE and VE, however, except for SA-Vlc-Abd, angular velocity maxima were higher in RE compared with the velocity maxima in both VE and VEF. As expected the velocity maxima were higher in RE than in VE and VEF, and they were generally, except for SI-Vlc-Int and SI-Vlc-Ext, lower in VE compared with VEF for “*fitter”* objects (Figs. 3 and 4).

**Fig 5:**
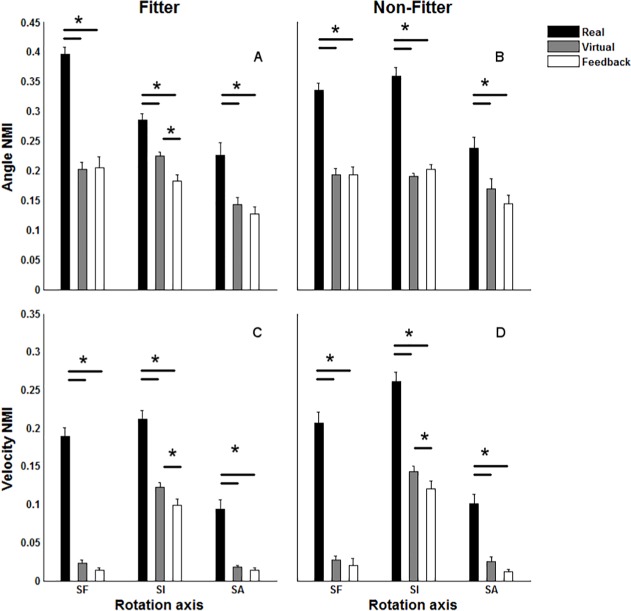
The mean and standard error of normalized mutual information (NMI) between the angular trajectories and velocity profiles within real environment and a representative trajectory, i.e., the median of traces across all subjects (Real). For kinematic trajectories of an arbitrary subject (*S_i_*), we computed *NMI*(*RE_S_i__,RE_S¯_*) where (*S¯*) indicates the representative trajectory across the subject pool. NMI between the virtual and virtual with force feedback trajectories on one hand and their corresponding trajectories within the real platform on the other hand (Virtual and Feedback) *NMI*(*RE_S_i__,VE_S_i__*) and *NMI*(*RE_S_i__,VEF_S_i__*), respectively. The figures in each column illustrate the outcomes for each of the object types (“fitter” and “non-fitter”). SF, SI and SA stand for shoulder flexion, internal rotation and abduction, respectively. * indicate a significant pairwise comparison between Real, Virtual and Virtual environment with feedback (Feedback) (p<0.05).

The platform or its interaction with the object type had a significant effect on the NMI, CC, and RMS.Diff of angular trajectories and angular velocity profiles. NMI and CC were largest in RE among the platforms whereas RMS.Diff was lowest in RE (Fig. 5 and Table 1). Further, NMI and CC were lower in VEF compared with VE for SI whereas RMS.Diff revealed an opposite pattern. NMI detected a significant difference between VE and VEF in terms of the SI-Vlc-NMI.

**Table 1:** The mean and standard error of cross-correlation (CC) and root mean square of the difference (RMS. Diff) across the participants and the cycles for shoulder flexion (SF), internal rotation (SI), abduction (SA) angle and angular velocity.

Platform		RE	VE	VEF	RE	VE	VEF
Object type	angle	CC	CC	CC	RMS. Diff	RMS. Diff	RMS. Diff
Fitter	SF	**0.83 (0.04)**	0.1 (0.04)	0.11 (0.05)	**16.7 (1.0)**	**35.1 (1.2) $**	49.8 (2.9)
	SI	**0.72 (0.03)**	**0.18 (0.04) $**	0.02 (0.04)	**10.6 (1.0)**	**16.5 (0.9) $**	25.9 (1.9)
	SA	**0.58 (0.05)**	0.1 (0.05)	0.14 (0.06)	**13.7 (1.4)#**	17.4 (1.6)	21.4 (3.2)
Non-Fitter	SF	**0.75 (0.04)**	0.11 (0.04)	0.16 (0.05)	**13.8 (1.0)**	32.4 (1.4)	34.6 (3.1)
	SI	**0.83 (0.02)**	**0.29 (0.04) $**	0.13 (0.04)	**9.3 (0.8)**	**16.5 (0.9) $**	29.6 (1.5)
	SA	**0.60 (0.05)**	0.01 (0.05)	0.03 (0.06)	**16.1 (1.8) #**	19.5 (2.1)	20.1 (3.1)
	Velocity						
Fitter	SF	**0.65 (0.02)**	0.0 (0.03)	0.02 (0.03)	**54.0 (2.8)**	76.2 (3.0)	81.2 (9.5)
	SI	**0.51 (0.03)**	0.08 (0.02)	-0.02 (0.02)	**40.0 (1.8)**	50.5 (2.6)	52.8 (3.0)
	SA	**0.37 (0.03)**	0.02 (0.02)	0.03 (0.02)	**46.0 (2.5)**	52.2 (2.9)	57.2 (7.9)
Non-Fitter	SF	**0.67 (0.02)**	-0.03 (0.04)	0.02 (0.05)	**41.8 (2.7)**	63.2 (3.2)	57.6 (2.4)
	SI	**0.68 (0.02)**	0.11 (0.02)	0.01 (0.04)	**33.9 (1.3)**	51.0 (1.9)	50.4 (2.4)
	SA	**0.41 (0.03)**	0.03 (0.04)	0.0 (0.04)	**47.8 (2.3)**	54.5 (2.7)	52.5 (2.9)

RE, VE and VEF represent “Real”, ‘’Virtual” and “Virtual with force feedback” platforms, respectively and “fitter” and “non-fitter” determine the object type. The CC and RMS. Diff within RE represent the mean of CC and RMS.Diff, the angular trajectories and velocity profiles for an arbitrary participant (*S_i_*) and a representative trajectory, i.e., the median of traces across all subjects (*S¯*). Thus, CC and RMS. Diff within RE indicate CC or *RMS.Diff*(*RE_S_i__,RE_S¯_*). Whereas CC and RMS. Diff within VE and VEF were between the kinematic trajectories in virtual and virtual with force feedback platforms on one hand and their corresponding trajectories within the real platform on the other hand, i.e., CC or *RMS.Diff*(*RE_S_i__,VE_S_i__*) and CC or *RMS.Diff*(*RE_S_i__,VEF_S_i__*), respectively. The bold fonts in RE columns indicate significant difference between RE and both VE and VEF.

# indicates that this difference was significant only between RE and VEF.

$ indicates a significant difference between VE and VEF.

**Fig 6:**
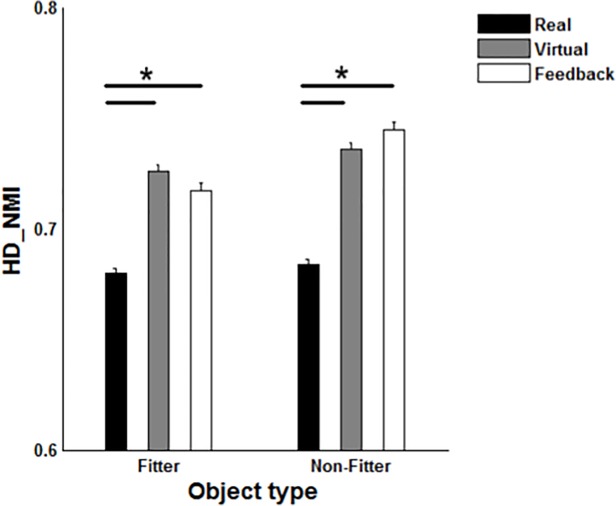
The mean and standard error of normalized mutual information within real, virtual and virtual with force feedback environment (Real, Virtual and Feedback) for fitter (A) and non-fitter objects (B). For an arbitrary subject (*S_i_*), we computed inter-subject similarity *NMI*(*RE_S_i__,RE_S¯_*) where *S_i_* and *S¯* indicate the electromyography map for i-th subject and the representative electromyography map across the subject pool, respectively (Real). The intra-subject similarity was computed across the virtual platforms *NMI*(*RE_S_i__,VE_S_i__*) and *NMI*(*RE_S_i__,VEF_S_i__*), for virtual and virtual with force feedback (Feedback) environments, respectively. * indicate a significant pairwise comparison between Real, Virtual and virtual environment with feedback (Feedback) (p<0.05).

As opposed to the kinematic trajectories, the HD-NMI was generally lowest in the RE platform (Fig. 6). The type of platform had a significant effect on the ratings of perceived exertion (P = 0.002). The subjects reported a higher score in VEF (2.4 (0.4)) compared with the other platforms (1.0 (0.2) and 0.9 (0.2) for RE and VE, respectively).

The interaction of platform × object type had a significant effect on the cycle time (P<0.001). The cycle time was shortest in RE and longest in VEF with “*fitter*” (2.8 s (0.1), 4.7 s (0.3) and 16.8 s (3.3) for RE, VE, and VEF, respectively). The cycle time was shortest in VE and longest in VEF with “*non-fitter*” (2.5 s (0.1), 2.0 s (0.2) and 4.8 s (0.9) for RE, VE, and VEF, respectively).

### Effect of Task Attributes

The cycles with “*non-fitter*” objects required higher SF angles from the reference standing posture compared with “*fitter*” objects, but the angular velocity was higher for “*fitter*” objects. CC of SI angle was lower for “*fitter*” objects compared with “*non-fitter*” objects in all platforms, but CC of SI angular velocity was lower for “*fitter”* compared with “*non-fitter”* objects in RE only. Similarly, SI-NMI and SI-Vlc-NMI in RE were lower for “*fitter*” compared with “*non-fitter”* objects. However, the SF-NMI in RE and SI-NMI in VE were higher in “*fitter*” than in “*non-fitter*” cycles. The RMS. Diff for both angle and angular trajectories was generally larger in “*fitter”* compared with the “*non-fitter”* cycles, but it was lower in terms of SI for “*fitter”* cycles in VEF. HD-NMI was lower for “*fitter*” than for “*non-fitter*” objects in all the platforms.

### The Bootstrap Resampling

As can be seen from Table 2 for angular trajectories as well as angular velocity profiles, the between subject variance component was larger than the between permutation variance component (16 and 17 out of 18 combinations of platforms, object types and the three shoulder rotation angles for angular trajectories and angular velocity profiles, respectively).

**Table 2:** The variance components of computed normalized mutual information (NMI) resulted from bootstrap resampling applied to the permutation of concatenation order of shoulder flexion (SF), internal rotation (SI) and abduction (SA) angle and angular velocity.

Platform	Object type	angle	BS	SE	WS	SE	Ratio
RE	Fitter	SF	1.0e-3	4.0e-4	6.0e-4	5.5e-5	1.7
		SI	1.5e-3	5.5e-4	3.2e-4	2.3e-5	4.7
		SA	8.0e-4	3.0e-4	4.7e-4	4.2e-5	1.7
	Non-Fitter	SF	2.7e-3	9.9e-4	4.0e-4	3.7e-5	6.7
		SI	1.4e-3	5.3e-4	2.9e-4	2.7e-5	4.8
		SA	1.2e-3	4.6e-4	3.3e-4	3.0e-5	3.6
VE	Fitter	SF	1.6e-4	5.9e-5	9.9e-5	9.0e-6	1.6
		SI	4.6e-4	1.7e-4	9.5e-5	8.7e-6	4.8
		SA	7.6e-5	2.9e-5	6.3e-5	5.8e-6	1.2
	Non-Fitter	SF	2.6e-4	1.0e-4	2.2e-4	2.0e-5	1.2
		SI	7.4e-4	2.7e-4	9.7e-5	8.8e-6	7.6
		SA	1.9e-4	7.4e-5	8.9e-5	8.2e-6	2.1
VEF	Fitter	SF	1.2e-4	5.7e-5	1.8e-5	2.1e-6	6.7
		SI	6.9e-4	3.2e-4	3.4e-5	3.9e-6	20.3
		SA	1.2e-4	5.6e-5	2.2e-5	2.5e-6	5.5
	Non-Fitter	SF	1.4e-4	7.0e-5	9.2e-5	1.0e-5	1.5
		SI	9.0e-4	4.3e-4	1.2e-4	1.4e-5	7.5
		SA	3.6e-5	1.9e-5	4.8e-5	5.5e-6	0.7

BS and WS represent the between-subject variance components and Ratio represents their ratio. SE represents the standard error of estimated variance components. RE, VE and VEF represent “Real”, “Virtual” and “Virtual with force feedback” platforms and “fitter” and “non-fitter” determine the object type.

## Discussion

The kinematics of the shoulder joint and the spatial activity of the upper trapezius were assessed during performance of a simulated assembly task on virtual platforms with and without force feedback and compared with the corresponding biomechanical parameters in a real environment. The measurements included the kinematics of the shoulder joint in 3D and the spatial pattern of the upper trapezius EMG activity. Further, the precision demand (“*fitter*” objects) generally resulted in increased kinematic maxima, lower EMG and lower inter and intra-subject similarity; however, this effect differed depending on the platforms. The CC and NMI were used as indices of similarity while RMS. Diff represented an index of variation of biomechanical responses which were used to compare the virtual platforms.

### Comparison of the Platforms

Although movement performance could be described by assessment of changes at the endpoint level, the present study only focused on the shoulder girdle because of the high prevalence of musculoskeletal disorders in this body region [45].

The comparison of kinematic properties during performance of a real motor task and a similar virtual task has previously been carried out in terms of range of movement and maximum velocity [8]. A similar range of trunk movements has been reported for virtual and real platforms but higher maximum velocity has been found in real environments than virtual environments [8]. The present results are in accordance with Whitman and co-workers (2004) [8] even though the maximum angles in RE were also different from those in VE in terms of SF-Flx, SA-Abd, and SI-Ext (see Fig. 3).

The observed differences in terms of maximum angles are also in agreement with Hu and co-workers (2011) [9], who found a smaller elbow angle within the VE platform compared with RE. For “*fitter”* objects the maximum angular velocities in VEF and RE were closer to each other compared with those in RE and VE except for SA-Vlc-Abd (see Fig. 4). As a general finding, the maximum velocity in RE was higher than in VE and VEF even though this was not observed for SI-Vlc-Int and SI-Vlc-Ext.

In this study CC and NMI were used as linear and nonlinear outcomes assessing inter-subject similarity in RE and intra-subject similarity in VE and VEF. Conversely, RMS. Diff was used to quantify inter-subject variation in RE and intra-subject variation in VE and VEF. CC and NMI also resulted in detection of greater inter-subject similarity in RE compared with intra-subject similarity across the virtual platforms. RMS. Diff as an index of variation showed an opposite pattern as compared with the similarity indices as expected. This means that inter-subject variation in RE was lower compared with intra-subject variation across the virtual platforms. Although the outcomes of CC, NMI and RMS. Diff were consistent, NMI was the only index detecting a difference between intra-subject similarities in VE compared with VEF in terms of angular velocity of the shoulder joint. This emphasized that nonlinear measures like NMI are capable of depicting changes in the dynamics of the kinematics time series. However, the application of NMI to a non-rhythmic signal may cause computational instability due to a large estimation variance caused by poor representation of the probability distribution function in the collected sample. To account for such limitations and increase the size of the samples involved in the estimation of NMI, we concatenated the kinematic trajectories before the NMI was estimated between the kinematic trajectories, and to verify whether this approach caused a build-up in estimation variance, we performed a bootstrap resampling technique to arrive at an empirical estimation of the variance components between and within subjects. We found that the between subject variance component was larger than the within-subject component (Table 2). This indicated that the adopted procedure did not result in an excessive estimation variance underlining that NMI can detect kinematic differences between RE and VE or VEF.

Contrary to our hypothesis, we found that the kinematic trajectories were more similar between the participants performing the task in RE compared with the similarity of kinematic trajectories belonging to a single participant working in different platforms. This is an important finding since a reliable evaluation of the biomechanics in VR platforms requires that the intra-subject similarity of the biomechanical responses across platforms is comparable with the inter-subject similarity of the biomechanical responses of the real work platform. A similar approach has previously been applied in ergonomics studies where the ratio between inter- and intra-subject variability has been used to contrast different working conditions [22,46]. It seems unlikely that the gradual adaptation of the participants to the task would result in a systematic bias to our results at the within-subject level because the participants performed the task in a randomized balanced order across the platforms. Additionally, if the adaptation level is assumed to be different across subjects, the between-subject variance increases and, in turn, results in lower inter-subject similarity. This supports our interpretation even further.

As another source of bias inherent to the current limitations of VR setups, the net external forces to the participant’s hand were not identical across the platforms. However, the shoulder movement patterns are reported to be quite stable over a range of small hand-loads (less than 2 kg) [47].

The similarity of the SI kinematic trajectories and velocity profiles between RE and VE was higher than the similarity between RE and VEF (Fig. 5). This appears to be in contrast with the expectation that haptic and tactile feedback will improve the fidelity of high-end VR systems [9]. However, there are still some technical issues in terms of incorporating force feedback in the design of a VR platform [2]. The co-localization of the haptic device in the current VEF platform was challenging and definitely needs further technical improvement. As the haptic device as well as the flystick partially occluded the scene to the user, it was necessary to introduce a 5 cm offset (see Fig. 1) between the virtual target and the real position of the end-effector of the device. It can be assumed that this offset could cause discrepancies between the kinematic trajectories across the real and virtual platforms. However, the offset was rather small and the same (see Section 2) applied to both VE and VEF conditions. Moreover, the coupling between the physical and the virtual positions of the device induced a perceptible delay (20–50 ms) between the real motion of the device and the displacement of the object on the scene. Further work is warranted to improve the standard control solution developed for the haptic device to enhance the interaction fidelity of the environment. Because of such limitations, the participants reported a higher rating of perceived exertion in VEF compared with the other two platforms. This difference can also be explained by the inexperience of the users since work experience interacts with the motor control of the shoulder girdle [34,48].

We expected that the inter- and intra-subject similarity of the EMG would indicate a trend similar to the kinematic trajectories, but contrary to our hypothesis, we observed that the inter-subject similarity between the EMG activation maps in RE was lower (lower NMI) than intra-subject similarity in VE and VEF platforms (Fig. 6). A similar contrast between EMG and kinematic pattern has previously been observed and explained by the notion of complexity trade-offs between the macroscopic (kinematics) and microscopic (EMG) levels of a control system [49].

The spatial pattern of muscular activity is of interest because it entails information such as the heterogeneity in the distribution of the muscle fibers recruited in performance of a task [50,51]. In accordance with our findings, high inter-subject variability has previously been reported in terms of spatial muscular activity [52]. Such participant-specific properties of the EMG maps could be partly related to changes in the spatial location of the recruited motor units depending on the level of exerted force, the adaptation to the task and the differences in muscular geometry. This study focused on the shoulder joint, and particularly the trapezius, not only because of its function during shoulder elevation, scapular rotation and arm elevation [53] but also because its typical anatomy and superficiality allowed application of HD-EMG. It is worth noting that investigation of muscle synergy [54] and its systematic changes across platforms is also relevant and feasible with the application of conventional bipolar EMG. However, the power of HD-EMG lies in its ability to provide a better spatial representation of the whole muscle, i.e., inhomogeneity in muscle recruitment [50]. Therefore, the application of NMI to HD-EMG maps in this study revealed a smaller inter-subject similarity within RE compared with the intra-subject similarity across the virtual platforms.

### Task Attributes

Comparison of precision demands showed that the cycles with “*fitter*” objects were generally characterized by lower deviation of shoulder angles from the reference standing posture, higher angular velocity and lower inter- and intra-subject similarity than “*non-fitter*” cycles.

Different shoulder angles and velocities in the cycles with “non-*fitter*” objects can probably be explained by the physical positioning of the disposal shelf and the work panel, which was placed at a lower height with respect to the shelf. They could also be caused by the physical configuration of the working setup as the shoulder was kept elevated to deal with the “*non-fitter*” objects. It is worth noting that this was the only direct comparison between the “*fitter*” and “*non-fitter*” objects and the rest of the comparisons were made to compare the similarity (NMI) of the corresponding kinematic trajectories in RE, VE, and VEF which handled the “*fitter*” and “*non-fitter*” objects. Similar comparisons were also made for muscular activity patterns within the platforms.

As mentioned above the different configuration of the work panel resulted in a more complex pattern of movement because of the higher precision demands dealing with “*fitter*” than “*non-fitter*” objects. Particularly, while the participants were passing the objects through the work panel, a relatively high level of precision was required. It is conceivable that the participants used a different motor strategy to perform this task, and the lower NMI or CC indicates higher inter- and intra-subject variability in terms of “*fitter*” objects. Further, longer cycle time was expected in VE and VEF than in RE because of the absence of reaction force from the rigid surface of the work panel in VE, the mechanical limitations of the haptic device in VEF and the lack of experience in virtual environments. Interestingly, the shortest cycle time was observed in VE with “*non-fitter*” objects. This probably implies that novice users could compensate for their lack of experience (after familiarization) provided that the task has a low precision demand. All in all, the present study emphasized the interaction effect of the precision demand and the biomechanical response in VR environments.

## Conclusion

A simulated assembly task was tested in RE, VE, and VEF. The biomechanical responses in VE and VEF environments were tested and compared with RE in terms of the spatial pattern of the trapezius activity and the 3D shoulder kinematics among novice users. The VE preserved the kinematic properties of the RE better than the VEF. Kinematic trajectories indicated a higher inter-subject similarity in RE compared with intra-subject similarity within the three platforms whereas the spatial pattern of the trapezius revealed an opposite pattern. The current study proposes a quantitative method to investigate VR platforms in terms of the biomechanics of a task. The proposed method does not provide an absolute criterion for the application or otherwise of VR platforms to ergonomics risk assessment. Nevertheless, the adopted approach offers a set of relative indices for assessing and comparing VR platforms with their real counterparts and provides a benchmark for evaluating modifications presented in VR platforms.

## Appendix

To compute NMI, the entropy H (average amount of information) of the observation was calculated. If X is a random variable, the entropy can be obtained from:

$$H\left(X\right)=-\sum p_{X}\left(x_{i}\right)\log\left(p_{X}\left(x_{i}\right)\right)$$ (1)

where *p_X_*(*x*) represents probability distribution function of X estimated using the histogram method and *p_X_*(*x_i_*) is the i^th^ bin of the normalized histogram [23]. The next step is to calculate the MI as defined by:

$$MI_{XY}=\sum_{i}\sum_{j}p_{XY}\left(x_{i},y_{j}\right)\log\left(\frac{p_{XY}\left(x_{i},y_{j}\right)}{p_{X}\left(x_{i}\right)p_{Y}\left(y_{j}\right)}\right)$$ (2)

where *p_XY_*(*x,y*) is the joint probability density function of X and Y. The upper bound of the MI calculated from (2) depends on the observation entropy. Therefore, MI varies between zero and minimum entropy of the signals. To establish a commensurable basis for comparison, a normalized version of the MI is presented as follows:

$$NMI=\frac{MI_{XY}}{\min\left\{H\left(X\right),H\left(Y\right)\right\}}$$ (3)

where NMI varies between 0 and 1. Furthermore, NMI = 1 if and only if X and Y carry identical information and zero when there is no shared information between X and Y [35]. This procedure can be generalized to two or more dimensional observations such as images.
